# Impact of college counselors’ spiritual guidance behaviors on Chinese college students’ employment anxiety: the chain-mediated roles of resilience and career calling

**DOI:** 10.3389/fpsyt.2025.1586101

**Published:** 2025-06-20

**Authors:** Gongjing Wang, Huadi Wang, Xueying Gu, Feifei Zheng

**Affiliations:** ^1^ School of Education Science, Nanjing Normal University, Nanjing, Jiangsu, China; ^2^ School of Geography and Planning, Chizhou University, Chizhou, Anhui, China; ^3^ College of Humanities and Arts, Nanjing Institute of Tourism and Hospitality, Nanjing, Jiangsu, China

**Keywords:** employment anxiety, spiritual guidance behaviors, resilience, career calling, spiritual leadership theory

## Abstract

**Objectives:**

Employment anxiety is a prevalent psychological issue among Chinese college students with far-reaching implications for both individual and societal well-being. College counselors play a vital role in students’ development by offering spiritual guidance through humanistic care, values shaping, and psychological support interventions. This study aims to investigate the multidimensional impact of college counselors’ spiritual guidance behaviors on Chinese college students’ employment anxiety, focusing on the mediating roles of resilience and career calling.

**Methods:**

Drawing on spiritual leadership theory, this study examined resilience and career calling as mediating factors. A cross-sectional survey involving 719 Chinese college students utilized the Spiritual Leadership Scale, Career Calling Scale, Resilience Scale, and Employment Anxiety Scale. Hierarchical regression analysis was conducted to develop a structural equation model elucidating how college counselors’ spiritual guidance behaviors influence students’ employment anxiety.

**Results:**

(i) Significant correlations were found among spiritual guidance behaviors, resilience, career calling, and employment anxiety. (ii) Path analysis demonstrated that resilience and career calling indirectly impacted employment anxiety through three pathways: namely, the independent intermediary role of resilience (indirect effect = -0.195), the independent intermediary role of career calling (indirect effect = -0.149), and the chain intermediary role of the two (indirect effect = -0.070).

**Conclusions:**

Studies show that college counselors play a crucial role in bolstering students’ resilience by offering spiritual guidance. This, in turn, nurtures a sense of career calling among students, leading to a systematic reduction in employment-related anxiety. These results support the implementation of a dual-path employment counseling model that integrates psychological and existential aspects.

## 1 Introduction

The issue of employment challenges among Chinese university students has gained prominence due to various factors, including the global economic slowdown, the expansion of higher education, reduced fiscal investment, and slow reforms in the higher education sector. The recent COVID-19 pandemic and increasing international competition have exacerbated the job market, leading to a phenomenon known as “employment involution” (1, 2). Employment involution refers to students’ repetitive and unproductive job-seeking behaviors, characterized by a lack of innovation and adherence to conventional practices—a phenomenon termed “path inertia.” This is evidenced by shifting educational values, limited employment opportunities, path dependency, and unclear career objectives (3).The uncertainty surrounding career prospects has heightened anxiety among university students regarding post-graduation employment (4–6). Elevated unemployment rates also increase the risk of mental health issues such as anxiety and depression among graduates (7). Research suggests that employment-related anxiety negatively impacts students’ psychological well-being, leading to reduced life satisfaction, impaired social adjustment, and hindered development (8). Therefore, investigating the mechanisms of employment anxiety among university students and proposing intervention strategies is crucial for addressing these challenges.

Employment anxiety results from a combination of external and internal factors. External influences primarily consist of social elements like social support and family upbringing (9–12). Internal factors mainly include demographic variables such as gender, age, institution, and major (7, 13), as well as individual psychological factors like self-esteem, self-efficacy, and psychological capital (11, 14–16). Internal psychological factors act as essential mediators through which external environments influence behavior. Individuals internalize external support into their affective and cognitive schemas, subsequently influencing their behavior. Research primarily focuses on the precursors of employment anxiety from theoretical standpoints in psychology, sociology, and economics. These studies investigate factors spanning individual psychological traits, social environmental elements, educational systems, labor market structures, and family background and support systems (17–19).

In recent years, interdisciplinary research has increasingly focused on examining the impact of spiritual leadership theory on college students’ employment anxiety. This theory emphasizes facilitating self-transcendence and life integration through humanistic care, psychological empowerment, and spiritual guidance. Scholars have investigated aspects such as college students’ employment anxiety and career confusion through the lenses of ideological and political education, as well as mentors’ spiritual guidance behaviors (20). The fundamental principle underpinning university counselors’ spiritual guidance behaviors is to foster individual inner growth, shape values, and construct meaning. This process involves reshaping students’ worldviews through humanistic care, enabling them to transition from passive anxiety management to proactive life planning. Such interventions not only mitigate immediate emotional distress but also cultivate long-term psychological resources to address anxiety by offering value orientation, integrating resources, and providing social support. To investigate the impact of university counselors’ spiritual guidance behaviors on college students’ employment anxiety, this study introduces two variables—resilience and career calling—and proposes tailored intervention strategies based on the research findings.

## 2 Literature review and research hypothesis

### 2.1 Theoretical basis

Employment anxiety goes beyond mere career considerations to delve into self-concept and life’s meaning, resonating with Spiritual Leadership Theory (SLT). Introduced by scholar Louis W. Fry in 2003, SLT integrates spirituality and leadership behaviors within a management framework. It is important to clarify that the “spiritual guidance” in this study refers to the secular aspects of meaning-making, purpose, values, and humanistic care, consistent with SLT, rather than religious guidance. This theory posits that spiritual leadership, which prioritizes individuals’ existential pursuits, aims to meet both personal and organizational spiritual needs to enhance intrinsic motivation, personal growth, organizational sustainability, and overall attitudes, cognition, emotions, and behaviors (21, 22). SLT redefines leadership from conventional “control and motivation” to “meaning-making and value resonance,” emphasizing the cultivation of a purpose-driven culture through vision, altruistic love, and hope/faith to achieve mutual prosperity for individuals and organizations (21). Specifically, spiritual leadership addresses employees’ spiritual needs by articulating an organizational vision, demonstrating care for subordinates, and reinforcing their beliefs and hope, thereby boosting their sense of calling and social connectedness, which subsequently impacts organizational commitment and productivity (21, 23). Research suggests that the inspirational and spiritual impact of spiritual leadership can motivate employees to exhibit improved work attitudes and behaviors (24).When individuals’ inner needs are met, intrinsic motivation is generated, leading to enhanced performance.

Intense competition in the job market has subjected college students to significant career development challenges, leading to heightened employment anxiety and negative career outlooks (25). Research suggests that factors such as insufficient career guidance, distorted thinking, social comparisons, and job uncertainty are closely linked to this anxiety among students (5, 26, 27). SLT, a comprehensive framework merging psychology, education, and behavioral science, aims to enhance individuals’ self-awareness and behavior through intrinsic motivation, values, and interventions (28). Humans are viewed as holistic beings comprising physical, emotional, intellectual, and spiritual dimensions, with spirituality being a vital life force that distinguishes them from other organisms (29). Examining how college counselors’ spiritual guidance can mitigate students’ employment anxiety offers a fresh perspective. This study employs SLT to introduce two pivotal variables—resilience and career calling—to establish a mediation model. The objective is to explore the psychological mechanisms through which counselors’ spiritual guidance influences college students’ employment anxiety.

### 2.2 Research hypothesis

#### 2.2.1 Spiritual guidance behavior and employment anxiety

Employment anxiety is a prolonged and heightened psychological condition marked by apprehension and stress in the context of job-related circumstances, accompanied by physiological and behavioral alterations (30). It stems from an individual’s adverse cognitive evaluation of employment objectives, procedures, and results, culminating in a state of agitated emotions. This concept is defined as a distinct form of transient anxiety induced by employment-related issues (31). Spiritual guidance is an intervention strategy that emphasizes stimulating intrinsic motivation, orienting values, and constructing meaning. It is designed to enhance individuals’ self-development and balance between internal and external factors in career advancement and stress management through deep psychological, emotional, and cognitive engagements. Its primary feature involves going beyond conventional skill training and providing information, prioritizing individuals’ spiritual requirements and enduring welfare (22).

Situations play a crucial role in driving the manifestation of personality traits (32). College counselors operationalize abstract spiritual principles into practical frameworks using five key situational dimensions: value orientation, psychological empowerment, behavioral support, cultural adaptation, and dynamic tracking. The fundamental concept involves establishing a “buffer zone” between stressors and individuals to facilitate the amalgamation of meaning-making and agency in students’ career decisions, psychological well-being, and social integration. This is achieved through the provision of resources, cognitive restructuring, and social support, effectively mitigating employment-related anxiety among college students (33). Amid prevalent employment concerns, college counselors offer spiritual guidance that operates synergistically across six dimensions: cognition, resources, psychology, values, behavior, and culture. This synergy leads to a combined effect of stress reduction, motivation enhancement, and capacity building. Counselors play diverse roles as cognitive reconstructors, resource facilitators, psychological mentors, value advisors, behavioral instructors, and cultural mediators. Their primary effectiveness lies in converting external job-related stress into internal drive for personal development, thereby transitioning from reactive coping with anxiety to proactive adjustment. At the core of college counselors’ spiritual guidance is the transformation of career decisions from being externally coerced to internally significant. By equipping students with practical tools and support, they enable individuals to adapt to change and stress in a resilient manner following adversities, stressful circumstances, or setbacks (34), ultimately diminishing employment-related anxiety. Thus, we posit Research Hypothesis 1:

Hypothesis1 (H1): College counselors’ spiritual guidance behaviors exert a significant negative impact on Chinese college students’ employment anxiety.

#### 2.2.2 Mediating role of career calling

Career calling is the perception of being summoned beyond oneself to fulfill one’s role by aligning vocation with life purpose and meaning, with a primary emphasis on altruistic values and goals (35). This concept encompasses dimensions including transcendent summons, purposeful work, and prosocial motivation. The process of career calling is dynamic and comprises three states: calling search, calling perception, and calling realization (36–38). It represents a high-level vocational outlook where individuals feel destined to pursue a specific career path, often emphasizing spiritual pursuits. Career calling not only has existential significance but also offers valuable insights into various organizational phenomena (39). The evolution of new career paradigms has significantly influenced individuals’ vocational values. Beyond traditional motivators like financial incentives and career advancement, individuals now place greater importance on self-worth and the sense of purpose derived from their careers when making career choices and progressing professionally (40). As a result, career calling has garnered increasing attention in the field of organizational management.

College counselors play a crucial role in guiding students towards exploring career paths by providing spiritual support. This involves creating an inspiring vision, helping students embrace uncertainties in career development, establish career beliefs, and fostering intrinsic motivation for pursuing their calling. By addressing students’ needs for respect, belonging, and self-actualization, counselors assist in students’ perception of career meaning, development of a sense of mission, and ultimately achieving a sense of calling, thereby progressing towards realizing their career aspirations. Career calling signifies a deep passion for a specific professional field, characterized by positive emotions, and plays a vital role in career decision-making and development, offering a unique approach to alleviate employment anxiety among university students (41). Drawing from self-determination theory, individuals with strong intrinsic motivation tend to have clearer goals and engage in persistent self-motivation to attain them (42). Embracing a career calling enhances intrinsic motivation, aiding individuals in clarifying their professional objectives and persistently working towards them, leading to positive outcomes such as increased self-confidence and self-efficacy (43). Furthermore, embracing a career calling enhances career certainty, as individuals guided by inner spiritual pursuits during career selection exhibit higher career maturity (43, 44) and greater decisiveness in career decision-making. Acting as a guiding principle in life, a career calling is linked to personal health, well-being, and a sense of purpose, resulting in positive altruistic outcomes. Students perceiving a career calling are more likely to develop a clear understanding of their career goals, make decisions aligned with their self-concept, and enhance their sense of career significance. This motivates them to actively explore career opportunities, improve their job-seeking skills, and resilience to stress, ultimately reducing employment-related anxiety.

In essence, college counselors address students’ intrinsic needs by providing spiritual guidance, including vision inspiration and meaning-making, to enhance their spiritual well-being. This process aids students in recognizing their vocational calling, fostering prosocial motivation, and nurturing a sense of work-related significance (45). As a result, students can clarify their career objectives, improve their self-concept, and promote favorable career psychology, ultimately shaping their expectations regarding career outcomes. Therefore, we posit Research Hypothesis 2:

Hypothesis 2 (H2): Career calling plays a mediating role in the impact of college counselors’ spiritual guidance behaviors on Chinese college students’ employment anxiety.

#### 2.2.3 Mediating role of resilience

Resilience is the capacity of individuals to withstand and even thrive in the face of significant stress or adversity (46). Positive psychology suggests that resilience signifies an individual’s inherent ability to quickly recover from setbacks, failures, engage in proactive behaviors, and manage increasing responsibilities (47). In the context of work, resilience is commonly defined as the capability to adapt to changing work environments and bounce back from career challenges (34, 48). Trait activation theory proposes that individuals possess latent traits that can be activated in appropriate circumstances, leading to corresponding behavioral responses. Research indicates that boosting self-esteem, self-efficacy, and social support can enhance resilience (49). Studies show a positive association between resilience and a proactive life stance, the pursuit of life purpose, and a negative correlation with perceived barriers to employment (50). College students who employ positive coping strategies and rational academic attributions tend to exhibit higher levels of resilience (51). College counselors who engage in spiritual guidance activities create nurturing, altruistic, and learning-focused environments for students. By offering vision, belief, and hope, these behaviors contribute to students’ sense of life purpose, thereby bolstering their resilience (52).

Resilience is a critical individual trait influencing emotional responses and mental health maintenance. Studies have consistently shown a strong inverse relationship between resilience and anxiety and depression, indicating that individuals with higher resilience tend to experience lower levels of these negative emotions. Furthermore, individuals with greater resilience demonstrate enhanced abilities to counteract the detrimental effects of adverse circumstances on their overall well-being. The compensatory model of resilience suggests that resilience acts as a protective factor for mental health, buffering the adverse impacts of stressors on individuals (53). Pietrzak et al. have also observed that individuals with high resilience possess ample psychological resources, enabling them to effectively manage negative emotions and stressful situations while displaying robust social adaptability (54). Moreover, research has shown that resilience can effectively moderate the link between negative life events and an individual’s mental health status (55). For college graduates, high resilience can help maintain mental well-being and reduce the psychological toll of employment pressures by employing effective coping strategies, thereby predicting lower levels of employment-related anxiety. Building upon this premise, we posit Research Hypothesis 3:

Hypothesis 3 (H3): College students’ resilience plays a mediating role in the impact of college counselors’ spiritual guidance behaviors on their employment anxiety.

#### 2.2.4 Chain mediation of resilience and career calling

To increase the likelihood of recognizing a career calling, individuals need not only clear self-awareness but also the corresponding skills to actualize this awareness (56). Resilience plays a vital role as an indicator of an individual’s available resources, providing them with strong psychological and behavioral abilities to navigate challenges encountered in perceiving, pursuing, and embodying a career calling. Through this process, individuals are more likely to cultivate a profound sense of purpose in their professional lives, a fundamental aspect of a career calling (45). Therefore, resilience contributes significantly to enhancing an individual’s level of experiencing a career calling. Building on this premise, the following hypothesis is posited:

Hypothesis 4 (H4): College students’ resilience and career calling play a chain mediating role in the impact of college counselors’ spiritual guidance behaviors and their employment anxiety.

**Figure 1:**
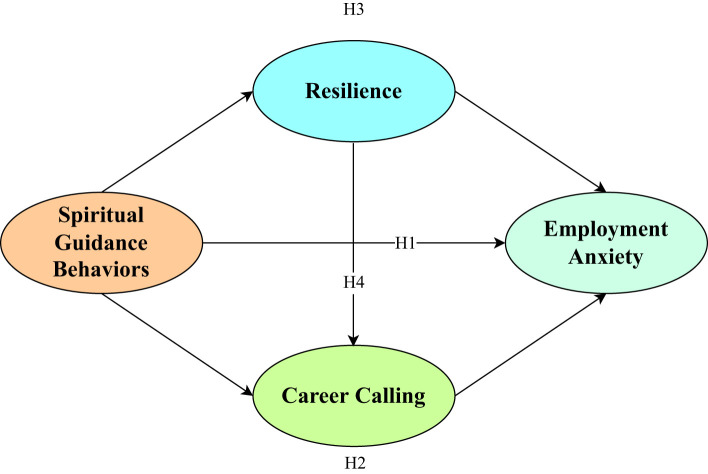
Hypothesis model.

Based on the abovementioned theoretical analysis and literature combing, a research hypothesis model was constructed, as shown in **Figure 1**.

## 3 Materials and methods

### 3.1 Participants

This research utilized a stratified cluster sampling approach to gather survey responses from 750 university students in China via the online platform Wenjuanxing. Collaboration with school officials facilitated the distribution of the questionnaire. Participants were briefed on the study’s aims, assured of the confidentiality of their personal data, and their voluntary participation was emphasized before the survey commenced. To mitigate duplicate submissions on Questionnaire Star and uphold data authenticity and validity, we employed several technical measures. Initially, respondents were mandated to furnish their mobile phone numbers. Subsequently, a single-choice logic question was integrated at the questionnaire’s outset. Additionally, a time threshold was enforced to filter out spurious responses. Thirty-one invalid samples were excluded, yielding a valid response rate of 95.9% (719 out of 750). The final sample size of 719 satisfied the criteria for conducting SEM analysis. The demographic distribution profile of the sample is detailed in **Table 1**. The study was carried out in May 2024.

**Table 1:** Characteristics of the demographic distribution within the sample (N = 719).

Items	N	%
Genders		
Male	294	40.89%
Female	425	59.11%
School level		
General undergraduate universities/colleges	296	41.17%
Non- ‘Double First-Class’ universities	278	38.66%
Double First-Class universities	145	20.17%
Types of schools		
public	586	81.5%
private	133	18.5%
Grade		
First-year	141	19.61%
Second-year	192	26.7%
Third-year	243	33.8%
Final-year	143	19.89
Census register		
rural areas	437	60.78%
urban areas	282	39.22%
Only child		
Yes	239	33.24%
No	480	66.76%

### 3.2 Instruments

#### 3.2.1 College counselors’ spiritual guidance behaviors

The present study utilized the indigenous spiritual leadership scale, as devised by Cui et al. (57), encompassing four dimensions: harmony spirit, moral exemplar, meaning giving, and vision inspiration, totaling 17 items. Employing a 5-point Likert scale (1=strongly disagree; 5=strongly agree), the scale measures the extent of spiritual guidance behaviors exhibited by university counselors, with higher scores denoting greater manifestation of such behaviors.

#### 3.2.2 Resilience

Resilience was assessed utilizing the revised scale by Connor and Davidson (58), comprising 25 items across three dimensions: strength, optimism, and tenacity. Responses were recorded on a 5-point Likert scale ranging from 1 (strongly disagree) to 5 (strongly agree). Higher scores on the scale are indicative of increased resilience in college students.

#### 3.2.3 Career calling

Zhang et al. (59) introduced the career calling scale, consisting of three dimensions: altruistic contribution, guiding force, and meaning and value, encompassing a total of 11 items. Respondents rated items on a 5-point Likert scale ranging from 1 (strongly disagree) to 5 (strongly agree). Elevated scores on the scale denote a heightened sense of career calling in university students.

#### 3.2.4 Employment anxiety

The Employment Anxiety Scale, created by Zhang and Chen in 2006 (60), comprises 26 items categorized into concerns regarding job opportunities, absence of employment assistance, diminished self-assurance, and pressure from employment competition. Respondents rate items on a 5-point Likert scale ranging from 1 (strongly disagree) to 5 (strongly agree). Elevated scores on the scale signify heightened levels of employment anxiety in college students.

**Table 2** displays the Cronbach’s α coefficients for the four scales: 0.953, 0.864, 0.857, and 0.968. Notably, some indicators within the Spiritual Guidance Behavior Scale of College Counselors and the Employment Anxiety Scale slightly exceeded the conventional threshold (RMSEA < 0.08) but remained within acceptable ranges.

**Table 2:** Summary of results of structural validity and internal consistency reliability tests for each scale.

Scales	Confirmatory factor analysis						Internal consistency reliability
	*χ*²/df	RMSEA	NFI	IFI	TLI	CFI	Cronbach *α*
College counselors’ spiritual guidance behaviors	3.266	0.093	0.958	0.963	0.949	0.963	0.953
Career calling	2.457	0.045	0.953	0.972	0.957	0.971	0.875
Resilience	1.505	0.027	0.934	0.977	0.965	0.976	0.864
Employment anxiety	3.88	0.105	0.946	0.952	0.934	0.952	0.968

### 3.3 Data analysis

This research utilized SPSS 26.0 for conducting reliability and validity assessments, descriptive statistics, correlation and regression analyses. Confirmatory factor analysis was executed with AMOS 29.0. The mediation analysis employed the PROCESS plugin in SPSS 26.0, utilizing the bias-corrected percentile Bootstrap method.

## 4 Results

### 4.1 Test for discriminant validity of variables

Before testing the research hypotheses, this study performed confirmatory factor analysis (CFA) with AMOS 29.0 to assess the discriminant validity of the latent variables. **Table 3** displays that among various factor models, the four-factor model exhibited the most favorable fit indices (χ2/df =4.859, RMSEA=0.073, CFI=0.964, TLI=0.954), confirming the sound discriminant validity of the measurement model.

**Table 3:** Confirmatory factor analysis results.

Models	χ^2^/df	CFI	TLI	SRMR	RMSEA
four-factor model + CMV	3.652	0.977	0.968	0.039	0.061
four-factor model: SGB, R, CC, EA	4.859	0.964	0.954	0.041	0.073
three-factor model: SGB, R+CC, EA	9.964	0.913	0.893	0.112	0.112
two-factor model: SGB + R + CC, EA	12.570	0.885	0.862	0.13	0.127
one-factor model: SGB + R + CC + EA	33.858	0.668	0.608	0.117	0.214

*n*=719; SGB, Spiritual Guidance Behavior of College Counselors; R, Resilience; CC, Career Calling; EA, Employment Anxiety.

### 4.2 Common method bias test

This study utilized Harman’s single-factor test to assess common method bias. The unrotated exploratory factor analysis indicated that the initial factor explained 33.4% of the variance, falling below the critical threshold of 40% (61, 62). To address common method variance (CMV), a five-factor model was constructed, including a CMV factor. **Table 3** displays that the five-factor model did not significantly enhance model fit compared to the four-factor model (Δχ2/df=1.207, ΔCFI=0.013, ΔTLI=0.014, ΔSRMR=0.002, ΔRMSEA=0.012). If the original confirmatory factor analysis (CFA) model with correlated factors demonstrates notable improvements in fit indices following the addition of a method factor (e.g., CFI and TLI increases exceeding 0.1, RMSEA and SRMR decreases surpassing 0.05), it suggests the presence of substantial common method bias (63). Consequently, this study is not significantly affected by common method bias, enabling further statistical analyses.

### 4.3 Descriptive statistics and correlation analysis

**Table 4** presents the descriptive statistics and correlation analysis of the variables under examination in this study. The findings reveal significant positive correlations between the spiritual guidance behavior exhibited by university counselors and students’ resilience, as well as their sense of career calling. Conversely, a significant negative correlation is observed between spiritual guidance behavior and students’ employment anxiety. These results suggest that heightened spiritual guidance behavior from university counselors is linked to increased levels of career calling and resilience, and decreased levels of employment anxiety among students. Moreover, career calling demonstrates a significant positive correlation with resilience, while both career calling and resilience exhibit notable negative correlations with students’ employment anxiety.

**Table 4:** Descriptive statistics and correlations of all variables (n=719).

Variables	M	SD	1	2	3	4
1. Spiritual guidance behaviors	4.43	0.60	–			
2. Career calling	4.50	0.33	0.631**	–		
3. Resilience	4.40	0.34	0.695**	0.588**	–	
4. Employment anxiety	2.29	0.74	−0.528**	−0.537**	−0.527**	–

**p<0.01. 1 stands for Spiritual Guidance Behaviors, 2 stands for Career Calling, 3 stands for Resilience, and 4 stands for Employment Anxiety.

The robust correlation coefficients between the independent and dependent variables necessitate an investigation into potential multicollinearity concerns. **Table 5** displays variance inflation factor (VIF) values for the three independent variables, which are 2.267, 1.790, and 2.084, respectively, all falling below the critical threshold of 5. These findings confirm the absence of multicollinearity among the independent variables.

**Table 5:** Multicollinearity analysis of all independent variables(n=719).

Models	Unstandardized Coefficient		Standardized Coefficient	*t*	Significance	Collinearity statistics	
	B	Standard Error	Beta			Tolerance	VIF
(Constant)	8.387	.352		23.848	<.001		
Spiritual guidance behaviors	-.236	.055	-.192	-4.305	<.001	.441	2.267
Career calling	-.634	.089	-.282	-7.119	<.001	.559	1.790
Resilience	-.498	.094	-.228	-5.326	<.001	.480	2.084

Dependent variable is Employment anxiety.

### 4.4 Regression analysis and mediation effect testing

This study develops an analytical model rooted in theoretical hypotheses to investigate how resilience and career calling mediate the association between the spiritual guidance behaviors of college counselors and employment anxiety in college students. The findings presented in **Table 6** indicate a positive association between counselors’ spiritual guidance behaviors and college students’ resilience (β=0.693, p<0.001) as well as their career calling (β=0.630, p<0.001), while demonstrating a negative relationship with employment anxiety (β=-0.526, p<0.001), thus providing support for Hypothesis H1. Both resilience and career calling are inversely related to employment anxiety (β=-0.527, p<0.001; β=-0.534, p<0.001, respectively). In a regression model where resilience and career calling were simultaneously included to explore the link between counselors’ spiritual guidance behaviors and students’ employment anxiety, all three variables—spiritual guidance behaviors (β=-0.189, p<0.001), resilience (β=-0.278, p<0.001), and career calling (β=-0.235, p<0.001)—exhibited significant negative associations with employment anxiety. The diminished direct impact of spiritual guidance behaviors on employment anxiety suggests the presence of mediating effects, thus confirming Hypotheses H2, H3, and H4.

**Table 6:** Multilayer linear regression analysis results. (n=719).

Variables	Resilience	Career calling		Employment anxiety			
	Model 1	Model 2	Model 3	Model 4	Model 5	Model 6	Model 7
School level	0.017	−0.037	−0.042	0.038	0.015	0.044	0.032
Types of school	0.021	−0.006	−0.001	−0.045	0.026	0.043	0.048
Only child	0.021	−0.036	−0.037	0.089*	0.061	0.092*	0.084**
Census register	0.000	−0.052	−0.060	−0.074*	−0.096**	−0.069*	−0.089**
Grade	0.024	−0.018	−0.051	0.088**	0.095**	0.115**	0.089**
Genders	−0.026	0.005	0.009	0.019	0.031	0.014	0.014
Spiritual guidance behaviors	0.693***	0.630***		−0.526***			−0.189***
Career calling					−0.534***		−0.235***
Resilience			0.587***			−0.527***	−0.278***
R^2^	0.485	0.405	0.356	0.297	0.306	0.300	0.391
△R^2^	0.470	0.388	0.339	0.271	0.280	0.274	0.365

*p<0.05, **p<0.01, ***p<0.001.

**Figure 2:**
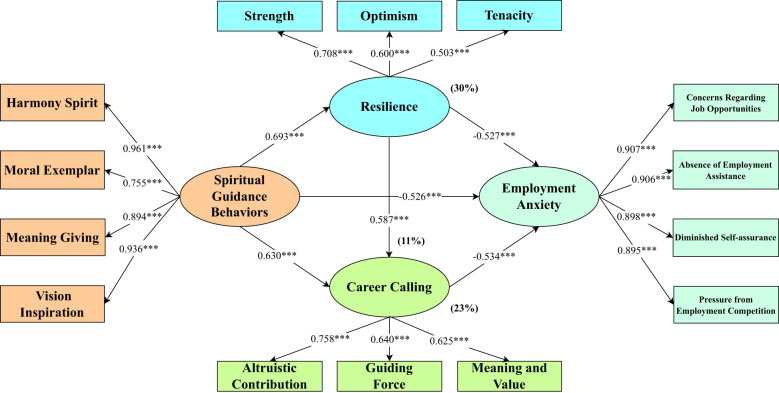
The theoretical model results of this study. ***p<0.001. The proportion of indirect effect is already in the parentheses.

The study revealed a significant total effect of college counselors’ spiritual guidance behaviors on students’ employment anxiety (Effect= -0.650, SE= 0.039, 95% CI [-0.726, -0.573]). Additionally, a direct and significant effect of college counselors’ spiritual guidance behaviors on students’ employment anxiety was observed (Effect= -0.236, SE= 0.055, 95% CI [-0.344, -0.128]), confirming Hypothesis H1. Past research has established that when the confidence interval does not encompass zero, the indirect effect can be affirmed (64). The findings indicated a significant mediating effect of resilience, with a Path 1 effect size of -0.195 and a 95% confidence interval of [-0.278, -0.090], explaining 30% of the total effect. This underscores the mediating role of students’ resilience in the association between college counselors’ spiritual guidance behaviors and employment anxiety, supporting Hypothesis H3. In Path 2, a significant mediating effect of career calling was observed, with an effect size of -0.149 and a 95% confidence interval of [-0.204, -0.103], explaining 23% of the total effect. This highlights that students’ career calling serves as a mediator in counselors’ spiritual guidance behaviors on employment anxiety, validating Hypothesis H2. Regarding the relationship between college counselors’ spiritual guidance behaviors and employment anxiety, validating Hypothesis H2. The sequential mediation of career calling and psychological resilience revealed an effect size of -0.070, with a 95% confidence interval of [-0.139, -0.027], excluding zero and explaining 11% of the total effect. This suggests that psychological resilience and career calling jointly play a crucial mediating role in linking spiritual guidance behaviors to employment anxiety. Therefore, Hypothesis H4 was upheld. The statistical analysis results from the PROCESS model are detailed in **Table 7**. **Figure 2** presents the test results of the research hypothesis model, which primarily comprises the path coefficients of the model and the effect size share.

**Table 7:** Bootstrap analysis of mediating effect significance test.

Name	Effect value	Boot SE	95% CI		Effect ratio (%)	*P*
			Boot LLCI	Boot ULCI		
Total effect	−0.650	0.039	−0.726	−0.573		0.000
Direct effect	−0.236	0.055	−0.344	−0.128	36	0.000
Total indirect effect	−0.414	0.048	−0.507	−0.321	64	0.000
Path 1	−0.195	0.048	−0.278	−0.090	30	0.000
Path 2	−0.149	0.026	−0.204	−0.103	23	0.000
Path 3	−0.070	0.029	−0.139	−0.027	11	0.000

Path 1: spiritual leadership behavior-resilience-employment anxiety; Path 2: spiritual leadership behavior-career calling-employment anxiety; Path 3: spiritual leadership behavior -resilience-career calling -employment anxiety.

**Figure 2** presents the test results of the research hypothesis model, which primarily comprises the path coefficients of the model and the effect size share.

## 5 Discussion

Within China’s higher education system, the spiritual guidance provided by university counselors significantly impacts the employment quality of college students in various dimensions. This issue is not only crucial for individual career development but also has implications for national talent strategies, social stability, and the educational mission of higher education institutions. This study expands the application of spiritual leadership theory beyond the traditional “leader-subordinate” relationship to the “counselor-student” dynamic by introducing two novel variables—resilience and career calling. A chain mediation model is developed to elucidate the complex pathways through which university counselors’ spiritual guidance influences students’ employment anxiety, thereby broadening the theoretical scope of spiritual leadership theory. Empirical evidence reveals that counselors’ spiritual guidance has a significant negative impact on students’ employment anxiety, with career calling and resilience acting as mediating factors. These findings offer valuable theoretical insights and practical implications for understanding and addressing employment anxiety, enhancing career education for college students, and improving the career guidance capabilities of university counselors.

### 5.1 Theoretical implications

This study expands the scope of SLT by transitioning it from organizational contexts to educational and mentoring environments. While previous research has primarily focused on applying this theory in workplace settings (28, 65), recent studies have started exploring its relevance in educational contexts (20, 66). In management literature, prior research has shown that spiritually guided behaviors can enhance employee creativity (67) and influence organizational citizenship behavior through mediating variables (68). SLT underscores the promotion of individual growth by activating intrinsic motivation (22, 68, 69). This study uniquely adapts SLT to educational settings, characterized by developmental objectives and distinct relational dynamics, as opposed to the power structures and performance-driven outcomes typical of organizational environments. In educational settings, where egalitarian dialogue is prioritized over power hierarchies, SLT emphasizes building trust between educators and learners through active listening, empathy, and spiritual resonance to foster co-growth. By delving into meaning, values, and intrinsic motivation, it supports comprehensive human development, aligning effectively with the requirements of educational contexts. Unlike corporate environments that emphasize measurable performance indicators, educational settings focus on the learning process and personal transformation. SLT underscores a “sense of journey,” embracing uncertainty and exploration to accommodate the non-linear paths of individual development. Through practices like mindfulness, reflection, and ritualistic activities, it enables learners to tap into their inner wisdom, facilitating profound transformation beyond mere skill acquisition. In educational contexts, spiritual guidance can incorporate non-religious spiritual practices such as promoting self-awareness, emotional regulation, empathy, and the exploration of life’s meaning, rather than imposing specific beliefs. This approach assists students in navigating identity formation, existential concerns, and related challenges.

This study conducted a cross-situational validation of SLT within the educational context, contributing to the existing research on this theoretical framework. While traditional research on educational leadership has primarily focused on administrative control and teaching competency (70), SLT highlights the importance of influencing students through adaptable methods like vision guidance and values cultivation (20), presenting a fresh perspective for educational leadership studies. The key theoretical advancements are as follows: Firstly, spiritual mentoring reconceptualizes the teacher-student dynamic as a collaborative exploration of significance and values. College counselors facilitate a shift from “managerial oversight” to “co-creation of meaning” by crafting visions, instilling a sense of purpose, and unlocking students’ inherent capabilities. Secondly, traditional student management often relies on external rewards or basic psychological needs, while spiritual mentoring focuses on motivations that surpass individual desires. Through modeling and storytelling, counselors steer students towards pursuits of higher-order values. Thirdly, spiritual mentoring defines spirituality as a deep connection with oneself, others, and the environment. Counselors combine “ethics of care” and “spiritual development” through existential conversations, mindfulness training, and transforming adversity into purpose. Fourthly, on an organizational level, the spiritual mentoring practices of university counselors break down one-way managerial relationships, fostering a community of shared values where management evolves into a realm of resonating values. Lastly, in response to the current trend of excessive technological emphasis in student management, spiritual leadership advocates for a recalibration of instrumental reasoning and value-based reasoning, integrating “spirituality” with traditional Chinese educational insights.

Future research should aim to integrate SLT with theories such as self-determination theory(SDT), conservation of resources theory(COR), career construction theory(CCT), and transformational leadership theory (TL)to develop a comprehensive intervention framework for reducing college students’ employment anxiety. Spiritual leadership emphasizes meaning, belonging, and purpose, while SDT focuses on autonomy, competence, and relatedness as drivers of intrinsic motivation. Employment anxiety arises from uncertainty, low self-efficacy, and lack of social support. By combining SDT with SLT, individuals can be empowered through intrinsic motivation and spiritual meaning to address and transform employment anxiety positively. COR suggests that individuals manage external stress by acquiring, maintaining, and accumulating resources, with resource loss leading to anxiety. Employment anxiety often results from the fear of resource depletion, while spiritual guidance can enhance resource acquisition capacity through hope and resilience. The sense of meaning provided by spiritual guidance can act as a psychological resource, buffering energy depletion under employment pressure. Altruistic behaviors can further foster a cycle of resource appreciation, breaking the cycle of anxiety and resource depletion. CCT focuses on career adaptability, self-concept integration, and meaning-making to address external uncertainty through agentic narrative. SLT, on the other hand, aims to activate intrinsic motivation to help individuals find meaning. By integrating career construction theory with spiritual leadership theory, a closed loop of motivation and capacity can be established to tackle the challenges of meaning deficiency and competence panic that contribute to employment anxiety. Transformational and spiritual leadership jointly influence students’ employment perspectives by integrating motivation and meaning. TL offers guidance and confidence via vision, individualized attention, intellectual stimulation, and idealized influence. In contrast, spiritual leadership meets profound spiritual needs related to purpose, meaning, and belonging. This combined approach tackles both superficial stressors and fundamental sources of anxiety among students.

### 5.2 Implications in practice

College counselors can effectively reduce college students’ employment anxiety by providing spiritual guidance. This process operates through two mediating factors: resilience and career calling. This approach not only offers immediate relief but also supports students’ long-term career growth. To enhance outcomes, counselors should move beyond conventional fragmented career guidance models. Instead, they should implement a systematic approach that integrates value orientation, psychological capital accumulation, and vocational meaning construction for sustained intervention in alleviating employment anxiety.

This study proposes enhancing the central role of spiritual guidance provided by college counselors to bring about a transformative shift in student career counseling, moving from a focus on imparting skills to fostering psychological empowerment. Counselors are advised to evolve from being mere administrative overseers to becoming mentors for personal growth. By incorporating spiritual guidance practices, counselors can elevate the quality and effectiveness of career counseling, ultimately enhancing students’ mindfulness (24). Conventional research on employment anxiety primarily examines external stressors (71, 72), while SLT focuses on addressing students’ spiritual needs to reduce anxiety by satisfying their sense of meaning and belonging. To begin, it is crucial to enrich the guidance provided by college counselors regarding students’ values. Spiritual guidance practices highlight the importance of counselors integrating career development guidance with the exploration of life’s meaning. Through individual consultations, themed group sessions, and other modalities, counselors should assist students in establishing clear professional values rather than solely emphasizing job-seeking skills. For instance, organizing “Career Values Workshops” can prompt students to contemplate questions like “What motivates my work?” and “How does my career contribute to life’s purpose?” Second, a structured framework for enhancing college counselors’ spiritual guidance competencies should be established. Universities ought to offer organized training in techniques such as narrative therapy and career coaching to empower counselors to effectively help students align their intrinsic values with career decisions. Incorporating “spiritual guidance competency” into counselor evaluations and allocating resources for specialized training are recommended strategies. Third, a comprehensive “trinity” support initiative should be devised to establish an integrated “psychological-career” support system for university students. Through spiritual guidance from counselors, students can reflect on their professional values; psychological counseling centers can provide resilience training; and career centers can facilitate vocational mission activities. This holistic approach enables students to progress seamlessly from constructing meaning to enhancing skills.

This study suggests that enhancing the spiritual guidance skills of college counselors can improve students’ resilience, activate their career calling, and reduce employment anxiety. Spiritual guidance not only addresses immediate anxiety but also promotes long-term career satisfaction and well-being by enhancing resilience and career calling perceptions (73). Initially, college counselors assist students in cultivating positive self-awareness, boosting resilience, and enhancing stress management through spiritual guidance. Students with higher resilience are more inclined to actively seek employment opportunities and adjust career expectations, ultimately lowering anxiety levels (50). By guiding students to align their career choices with personal values, counselors can mitigate anxiety resulting from haphazard job selection or external pressures (74). Moreover, counselors should establish a “ resilience growth profile” to monitor students’ resilience changes over time. Group counseling can bolster stress management skills for lower-grade students, while higher-grade students can benefit from reflective exercises based on real-life challenges encountered in academic or practical contexts. Offering emotional support through attentive listening, encouragement, and empathy (75) can alleviate feelings of isolation and despair, directly reducing anxiety levels. Furthermore, counselors should stimulate the impact of career calling by elucidating career significance and aligning personal calling through concrete actions. By arranging interviews with career role models and facilitating service-learning projects, students can better grasp the societal value of professions, translating abstract aspirations into tangible career objectives. Vocational assessment tools can identify students’ inclinations towards calling, enabling tailored support for those with a strong calling inclination. Additionally, utilizing digital platforms to share career narratives and organize industry-related activities (76, 77) can help students clarify career significance and uncover intrinsic professional value, thereby diminishing anxiety related to utilitarian job choices.

This study suggests that in the Chinese cultural context, counselors’ spiritual guidance plays a dual role as a “pressure relief valve” and a “transformer.” It aims to ease structural pressures from traditional values while converting cultural resources into psychological capital to address college students’ employment anxiety by fostering resilience and shaping career calling (78). To enhance resilience, counselors utilize value-based guidance to help students reframe employment pressure as meaningful life challenges, strengthening their ability to cope with setbacks. Addressing the cultural emphasis on “guanxi” (relationships), counselors provide personalized care to bridge the psychological gap created by the “face” culture, which may hinder students from seeking help actively and lead to feelings of isolation. To counteract the fear of career setbacks due to the prevalent “one-exam-determines-life” mindset, counselors can offer growth mindset training to help students redefine failure. In terms of shaping career calling, counselors can assist students in aligning personal interests with societal needs, tapping into the collectivist culture’s dedication spirit to reinforce a sense of professional purpose (79). Discussions on “neo-filial piety” can help alleviate career-choice anxiety arising from the pressure to pursue high-paying jobs for family honor. To mitigate employment anxiety, students should be steered away from “involution” (excessive competition) and the pressures associated with “face” (80). Engaging in critical dialogues on the cultural origins of “overcompetition” can aid students in establishing distinct career evaluation criteria. In response to societal expectations regarding “respectable professions,” counselors can utilize case studies to broaden students’ perspectives on “success.”

### 5.3 Limitations and future directions

The study utilized a self-report questionnaire for data collection, passing common method bias tests. To enhance precision, future research could collect paired multi-wave data (81, 82). College students’ employment anxiety, as a psychological experience, is influenced by various contextual factors. This study focused solely on college counselors’ spiritual guidance behavior, neglecting the impact of Chinese cultural background on the observed relationships. Future studies should consider variables such as students’ majors, economic backgrounds, psychological capital, stressful life events, and family characteristics to explore more effective intervention mechanisms (13, 83). The study employed a cross-sectional design with unequal sample sizes of upper- and lower-class students, potentially affecting the variation in employment anxiety levels across academic years. Future research could investigate measurement invariance to bolster academic rigor. Furthermore, the counselor-student relationship is a multifaceted, dynamic system. The study solely examined the one-way influence of university counselors’ guidance. Future research should consider multiple theoretical perspectives to delve into the mediating pathways and boundary conditions of how counselors’ spiritual guidance behavior impacts college students’ employment anxiety.

## 6 Conclusions

In essence, this study reveals that the spiritual guidance provided by university counselors significantly reduces college students’ employment anxiety. Resilience and career calling partially mediate the relationship between counselors’ spiritual guidance and students’ employment anxiety. By incorporating spiritual leadership theory into the counselor-student dynamic, this study not only offers a novel theoretical framework for addressing college students’ employment anxiety but also suggests avenues for enhancing the professional growth of university counselors. In the Chinese context, the spiritual guidance offered by university counselors serves not only as a tool for career advice but also as a crucial element in cultivating moral values through education. By integrating theory and practice, this approach can harmonize students’ personal and societal values, presenting a Chinese solution to the dual challenges of “employment difficulty” and “recruitment difficulty.” Future research should delve into the differential impacts of this guidance across disciplines and student cohorts to inform targeted university policies effectively.

## Data Availability

The original contributions presented in the study are included in the article/supplementary material. Further inquiries can be directed to the corresponding author.
